# 30. Weakness, pancytopenia and encephalopathy in a 66 year old woman: one diagnosis, two diagnoses, or more?

**DOI:** 10.1093/rap/rky033.021

**Published:** 2018-09-20

**Authors:** Kieran Sandhu, Mark Weatherall, Hannah Zacharias, Jasroop Chana, Malgorzata Magliano, Monika Hofer

**Affiliations:** 1 Rheumatology, Bucks Healthcare NHS Trust, Aylesbury, UNITED KINGDOM; 2 Neurology, Bucks Healthcare NHS Trust, Aylesbury, UNITED KINGDOM; 3 Academic Unit of Neuropathology, Oxford University Hospitals NHS Foundation Trust, Oxford, UNITED KINGDOM

**Introduction:** Colchicine is a commonly used medication in both primary and secondary care, most often in the acute management of crystal arthritides. It works by disrupting microtubule polymerisation and interfering with mitosis, thus impacting all dividing cells. Its therapeutic index is narrow; toxicity is dose-related and an extension of its mechanism of action. Constitutional and gastrointestinal upset occur commonly however there is also the potential for more severe systemic toxicity. This risk is increased in patients with hepatic or renal impairment, and in those taking CYP3A4 enzyme inhibitors such as clarithromycin and ketoconazole. Colchicine‐related neuromyotoxicity typically presents with proximal pain and weakness, with elevation of creatine kinase and transaminases. Colchicine-related myelosuppression leads to pancytopenia. Recognising these adverse effects early is vital because timely withdrawal of the drug can result in a full recovery whereas continued exposure invariably leads to further deterioration. We report a case in which both of these adverse effects occurred, in a patient with normal renal function, but who had taken clarithromycin alongside prolonged treatment with colchicine. The clinical course was complicated by transient encephalopathy. Subsequent investigations revealed a mutation in the nuclear *POLG* gene. We discuss whether the presence of an underlying mitochondrial disorder may have predisposed the patient to the development of colchicine toxicity, and review the approach to prescribing colchicine in patients at increased risk of toxicity.

**Case description:** A 66 year old lady presented with a two week history of pain and weakness in her upper and lower limbs on a background of a chronic 18 month illness consisting of diarrhoea, fatigue, recurrent rashes, myalgia, reduced appetite and weight loss. Her past medical history consisted of gout, congestive cardiac failure, type 2 diabetes mellitus, hypertension, primary hyperparathyroidism, transient ischaemic attacks, osteoarthritis of the knees and Cushing’s syndrome, for which she had a unilateral adrenalectomy two years previously. She had been suffering acute attacks of gout with increasing frequency and been unable to tolerate allopurinol due to side effects. Other medications included metformin, bisoprolol, clopidogrel, lansoprazole, losartan and furosemide. She had also taken a prolonged course of clarithromycin for four weeks, which was completed a few days prior to admission. There was no family history and she was previously independent, living with her husband. On examination she was alert and oriented, with normal cardiorespiratory and abdominal examinations. She had proximal weakness MRC grade 3/5 in her shoulders with less marked weakness distally at 4+/5 in her hands. Lower limb weakness was profound at MRC 1/5 for hip movements and 2-3/5 distally. The weakness was fixed with no clinical evidence of fatiguability. It was associated with myalgia, particularly of the thighs and upper arms. She had a complete absence of deep tendon reflexes and plantars were downgoing. There was distal impairment of sensation in the upper and lower limbs. Fundoscopy and cranial nerve examination were unremarkable. She had a macular rash over her upper arms and flanks. There was no synovitis, dilated nailfold capillaries or splinter haemorrhages. Blood pressure was 194/100, but she was afebrile and other vital signs were normal. Initial investigations revealed a total white cell count of 0.9 x10^9^/L with neutropenia of 0.2 x10^9^/L and lymphopenia of 0.4 x10^9^/L. Platelets were slightly low at 136 x10^9^/L but haemoglobin was normal. A blood film confirmed marked leucopenia but with no blasts or other diagnostic features. ALT was elevated at 270 IU/L, with slight elevation of ALP. A recent ultrasound had revealed fatty liver changes but no other structural liver pathology. Creatine kinase was markedly elevated at 5465 IU/L. Inflammatory markers, urea and electrolytes, clotting, thyroid function, troponin and vitamin D were all normal. Electrocardiogram and chest radiographs revealed no abnormalities, but urinalysis was positive for blood and protein with a raised protein creatinine ratio of 1950 mg/mmol. This had been raised previously and was thought to be related to diabetic nephropathy. The combination of myopathic and neuropathic signs on the background of a generalised systemic illness, with associated leucopenia, thromobocytopenia, transaminitis and raised creatine kinase, raised the possibility of colchicine toxicity. Further enquiries revealed that she had been taking colchicine at a dose of 500 micrograms twice daily in a five days on, seven days off pattern for two months prior to admission, and had continued to do so while taking clarithromycin. This was in addition to repeated shorter courses of colchicine prior to these two months. Colchicine was held on admission and she was treated supportively while further investigations were carried out. The clinical picture remained unchanged in the first few days, however her blood abnormalities improved rapidly: by day three her creatinine kinase was down to 978 IU/L, white cell count was up to 2.7 x10^9^/L with neutrophils of 1.1 x10^9^/L, platelets had normalised and ALT had come down to 130 IU/L. All of these continued to improve and normalised within a few days, other than the ALT which took a month. On day four of her admission she dropped her GCS to 7/15 (with some fluctuation). MRI brain revealed abnormal cortical high signal bilaterally affecting the frontal lobes with ill-defined signal in both temporal lobes. The spinal cord was normal. CSF analysis, extended autoimmune screen, tumour markers, viral serology, cortisol, immunoglobulins and multiple blood, urine and stool cultures were all negative. Transthoracic echocardiogram showed only mild systolic and diastolic dysfunction, and a CT chest abdomen pelvis showed no significant abnormalities. As she had been persistently hypertensive during the first few days of admission with systolic blood pressures ranging from 180-200 mmHg despite uptitration of antihypertensives, a working diagnosis of posterior reversible encephalopathy syndrome was made. Once better blood pressure control was achieved her encephalopathy resolved fully over five days, and repeat MRI showed resolution of the cortical high signal. She had started to show small improvements in power but her weakness remained severe. Further investigations were performed to elucidate the cause of her presentation. Nerve conduction studies revealed reduced amplitude of the compound muscle action potential, reduced or absent sensory potentials and absent F-responses in the lower limb. Electromyography showed active denervation in the distal muscles and small polyphasic motor units particularly in the proximal muscles. These results were consistent with a combination of severe distal axonal neuropathy and myopathy. An MRI scan of her thighs showed perifascial myositis adjacent to the right lateral femoral intermuscular septum involving the right vastus lateralis and right biceps femoris short head. A muscle biopsy revealed variably sized vacuolated muscle fibres and membrane-bound autophagic vacuoles with myofibrillar disarray and deposits of dark staining material. One COX-negative fibre and two possible ragged red fibres were also seen. The vacuolar changes strongly supported colchicine toxicity, and in conjunction with the suggestive clinical picture, this provided enough evidence to confirm the diagnosis. After withdrawal of colchicine, resolution of all blood test abnormalities occurred over days to weeks although the clinical recovery was somewhat slower. Management was supportive and focused on physiotherapy and rehabilitation. She was discharged 12 weeks after initial admission, by which point she had recovered her power to 5/5 in her arms and 4+/5 in her legs, allowing her to mobilise with a frame. She suffered a further acute attack of gout in her metatarsophalyngeal joints, which was managed successfully with a short course of prednisolone. She will be followed up by rheumatology to decide on alternative urate lowering therapy but should stay off colchicine in the future.

**Discussion:** Although generally considered safe, colchicine can produce significant and serious toxic effects. Our patient developed neuromyotoxicity and myelosuppression, both of which are rare but nonetheless well described. Nerve conduction studies, electromyography and muscle histology were consistent with previously reported cases. Early withdrawal of the drug usually leads to full recovery; this was seen in our case, although muscle power improved more gradually and recovery is ongoing, partly due to deconditioning. An additional interesting aspect of this case is, alongside typical colchicine-related vacuolar changes on histology, there were also two possible ragged red fibres which are characteristic of mitochondrial myopathies and 1 COX-negative fibre which may be found in mitochondrial or inflammatory myopathies. This prompted genetic analysis for major mitochondrial DNA mutations, which revealed she is heterozygous for the common *POLG* variant c.1760C>T p. (Pro587Leu). *POLG* is a gene that is associated with a group of disorders of mitochondrial DNA with overlapping phenotypes. Within their phenotypic spectrum, axonal neuropathies and myopathies have been reported. These disorders are autosomal recessive, with only homozygotes displaying symptoms. The question to consider, in light of the histological findings, is whether her carrier status either increased her risk of colchicine-related neuromyotoxicity or amplified the severity of her syndrome. It has been implicated that carriers of other *POLG* variants have an increased risk of valproate-induced hepatotoxicity, and it is not implausible that such an interaction could exist with colchicine-related neuromyotoxicity. Nevertheless even the interaction with valproate remains tenous and there is no conclusive evidence that our patient’s carrier status affected her colchicine toxicity syndrome. To investigate further, an interesting opportunity for future work would be to screen a cohort of patients with colchicine-related neuromyotoxicity and identify if they are enriched for common POLG variants and whether ragged red and COX-negative fibres occur in greater frequency in carriers.

**Key Learning Points:** Colchicine is a commonly prescribed drug and it is important to be aware of the risk factors that predispose to its toxicity, which can occur both in acute overdose and unintentionally with chronic use. Serum drug concentrations and therefore risk of toxicity are increased by hepatic impairment, renal impairment and concomitant use of CYP3A4 inhibitors most notably clarithromycin. The four-week course of clarithromycin in our patient was the likely trigger for her significant neuromyotoxicity although there was evidence of lower grade toxicity before this with persistent gastrointestinal symptoms. We advise using colchicine with caution in patients in whom these risk factors are present, and certainly avoiding long term use if possible. In our patient, alternative urate lowering therapy should have been used instead. If colchicine is to be used, clear limits should be set on dose and duration, and indeed dose-reduction algorithms have been proposed elsewhere. CYP3A4 inhibitors should be avoided, but if they are prescribed then colchicine should be held while taking them. Patients at significant risk of toxicity should be adequately counselled when starting colchicine and warned to report gastrointestinal side effects early as these may be a precursor to more serious adverse effects. This case highlights the need to be aware of colchicine’s wide range of toxicity and include it as differential for a wide range of presentations including myalgia, weakness and pancytopenia. Although recovery from both neurotoxicity and myelosuppression is usually complete, some cases have reported profound toxicity and death despite maximal supportive treatment. Therefore recognition and early withdrawal of the drug is vital and can be life-saving.

**Disclosure: K. Sandhu:** None. **M. Weatherall:** None. **H. Zacharias:** None. **J. Chana:** None. **M. Magliano:** None. **M. Hofer:** None.

